# Screening for latent tuberculosis infection by an Aboriginal Community Controlled Health Service, New South Wales, Australia, 2015

**DOI:** 10.5365/wpsar.2018.9.2.010

**Published:** 2019-12-17

**Authors:** Hannah Visser, Megan Passey, Emma Walke, Sue Devlin

**Affiliations:** a University Centre for Rural Health, Lismore, NSW Australia.; b North Coast Public Health Unit, Lismore, NSW Australia.

## Abstract

**Objective:**

Ongoing transmission of tuberculosis (TB) continues in Indigenous communities in New South Wales (NSW), Australia. In a pilot project, a Public Health Unit TB team partnered with an Aboriginal Community Controlled Health Service (ACCHS) in a community with a cluster of TB to augment screening for latent TB infection (LTBI) using interferon-gamma release assay (IGRA). This study examined screening data and programme outcomes at 12 months post hoc to advise practice and policy formulation.

**Methods:**

We conducted a retrospective, cross-sectional analysis of demographic and clinical data of ACCHS patients, stratified by IGRA testing status. Differences in sex and age distribution between the groups and cases of a genetically and epidemiologically linked TB cluster in Aboriginal people in NSW were assessed using non-parametric tests.

**Results:**

Of 2019 Aboriginal and Torres Strait Islander people seen by general practitioners during the study period, 135 (6.7%) participated in the screening. Twenty-four (17.8%) participants were IGRA positive. One person was diagnosed with active TB. Twelve participants received a chest X-ray at the time of the positive test, and six participants had an additional chest X-ray within 12 months. None commenced preventive treatment for LTBI.

**Discussion:**

ACCHS screening for LTBI reached individuals in the age group most commonly affected by TB in these Aboriginal communities. No conclusions can be made regarding the population prevalence due to the low screening rate. Further strategies need to be developed to increase appropriate follow-up and preventive treatment.

Tuberculosis (TB) is a major public health issue and a leading cause of death worldwide. (*1*) Despite the low incidence of TB in Australia (5.7 cases per 100 000 population in 2014), TB is still endemic in Indigenous communities. (*2*) Sociocultural factors and individual risk factors for infection contribute to ongoing TB transmission in Australian Indigenous communities. (*3*)

LTBI is infection with *Mycobacterium tuberculosis,* in the absence of clinical signs or symptoms of active TB. (*4*) The lifetime risk of TB reactivation is 5–10%, with most developing active TB in the first five years after infection. (*5*) Treating LTBI decreases the risk of active TB by 60–90%. (*6*) The most commonly used treatments for LTBI are six- or nine-month courses of isoniazid. (*7*)

The World Health Organization (WHO) set the goal of TB elimination by 2050 and initiated the End TB strategy in 2014. (*8*) In Australia, the National Tuberculosis Advisory Committee (NTAC) guidelines assist TB services with achieving programme targets. Indigenous Australians are included in the populations WHO and NTAC recommends for targeted testing and treatment for LTBI. (*8*, *9*)

There is no gold standard for the diagnosis of LTBI. The two tests currently used are the tuberculin skin test (TST) and interferon-gamma release assay (IGRA). While TST involves two encounters with specialized staff 48–72 hours apart, IGRA is a one-visit, whole-blood test that measures the immune response to antigens of *M. tuberculosis*. WHO recommends either TST or IGRA to test for LTBI. (*10*) At the time of this project, NTAC recommended TST to diagnose LTBI, but “IGRAs may be a preferred option where resources, distance or other factors make TST impractical to administer;” (*11*) IGRA was only funded under Medicare in Australia for immunocompromised patients. In 2017 the NTAC position statement was revised and now recommends “either TST or IGRA for the investigation of LTBI in most circumstances.” (*12*)

Since 2000, 48 genetically and epidemiologically linked cases of active TB (mycobacterial interspersed repetitive units [MIRU] pattern 23’3425153322) have been diagnosed in Aboriginal people. Many of these patients resided in an Aboriginal Community Controlled Health Service (ACCHS) catchment area in northern New South Wales (NSW), Australia. TB transmission has occurred despite implementation of TB control measures such as household contact tracing and community-based screening events using TST. (*13*)

With concerns about ongoing TB transmission and after consultations with Aboriginal people of the affected communities and TB expert committees, the local Public Health Unit (PHU) collaborated with the ACCHS in a pilot project to offer screening for LTBI and to provide preventive therapy.

To eliminate TB, the support of primary care providers who care for high-risk populations is essential. (*7*) Previous collaborations between ACCHS and NSW Ministry of Health highlight the benefits of partnerships. (*14*)

The overall objectives of the ongoing project include: to better understand the epidemiology of LTBI in the communities served by the ACCHS; to identify who is reached by the ACCHSs offering screening; to strengthen the partnership between the ACCHS and PHU TB Team; to raise TB awareness in the ACCHS setting; and to advise practice and policy formulation, including IGRA funding and incorporation of TB screening in annual health assessment in at-risk communities.

We describe a retrospective epidemiological analysis of data from the IGRA screening project with the following aims:

assess the reach of this model of TB screening for a rural Australian Aboriginal population;
compare the demographic characteristics of people in the project with the general ACCHS patient population and those in the TB MIRU pattern 23’3425153322 cluster in NSW; and
evaluate the TB screening outcomes at 12 months.

## Methods

General practitioners (GPs) and all clinical staff working for the ACCHS providing services in three Aboriginal communities in northern NSW received basic TB training and were encouraged to screen for TB and offer IGRA testing to their patients who presented for medical consultations at the central or one of the two outreach clinics between 3 June 2013 and 27 January 2015. The decision to test with IGRA was intended to treat those with TB as identified by the GPs and the PHU TB team (TB doctors, TB nurses and Aboriginal TB Community Engagement Officers). All patients were eligible for testing; however, testing was offered as part of a GP consultation; therefore, study inclusion was at the GP’s discretion.

The IGRA used in this project was Quantiferon TB Gold (QIAGEN GmbH, Hilden, Germany). Trained nurses at the ACCHS or at a private pathology service collected the samples, which were then sent to Brisbane for incubation and analyses within 16 hours.

Patients provided verbal consent to participate in the project, which included consent for sharing of their demographic and TB-related clinical data (IGRA, chest X-ray and sputum test results) with the PHU TB team. At the time of blood collection, nurses ensured that patients understood they were having an IGRA test, and patients provided signed consent to proceed.

Participants were questioned about active TB symptoms (cough, night sweats, weight loss). Participants’ data were recorded in their medical files at the ACCHS and in the Notifiable Conditions Information Management System (NCIMS) database, administered by NSW Health Ministry.

ACCHS doctors notified all participants of their results. If the IGRA test was positive or there were clinical concerns, participants were referred for a chest X-ray (at a private radiology service in the town of the main ACCHS clinic), sputum test for acid-fast bacilli (AFB) with smear, polymerase chain reaction (PCR) and culture (at a private pathology service) and to a TB specialist clinician to explore treatment options. The PHU TB team contacted all referred patients and offered a range of support services for further assessment and preventive TB treatment. When preventive treatment was declined or deemed inappropriate, the referring GP was informed and a repeat chest X-ray was offered to the patient 12 months after the positive IGRA result.

### Case definitions

We defined LTBI as a positive IGRA test in the absence of clinical manifestations of active TB based on symptoms, radiology and sputum test results.

The TB cluster cases are 37 epidemiologically linked, active TB cases in Aboriginal people in NSW with the MIRU pattern 23’3425153322 diagnosed between October 2000 and February 2015. Cluster cases diagnosed outside NSW and diagnosed after the study period were not included in the analysis.

### Data collection

We compared non-identifiable data retrieved from NCIMS and ACCHS. Demographic data including age, sex, Indigenous status and resident postcode of people screened with IGRA and people within the TB cluster were retrieved from NCIMS. Only Indigenous people were included in the study. Variables used for the outcome analysis of the people who screened positive for LTBI (IGRA result, sputum test results, chest X-ray at 0 and 12 months and TST results from previous screening) were also extracted from NCIMS.

Demographic data of patients presenting to the ACCHS between 3 June 2013 and 27 January 2015 were requested from the Executive Officer of the ACCHS and extracted from Medical Director software using the PenCat tool. (*15*)

### Data analysis

We undertook a descriptive analysis on the screening outcomes data. All data used were from people identifying as Aboriginal and Torres Strait Islander. Data from non-Indigenous people accessing the ACCHS were excluded. We compared the demographic characteristics of the ACCHS’s patient population, the individuals screened by IGRA and the individuals within the TB cluster cases in NSW. χ^2^ tests were used to assess differences in sex distribution between four groups (ACCHS patients, IGRA screening participants, IGRA screening participants who tested positive for LTBI and TB cluster cases). A non-parametric Kruskal–Wallis test and Mann–Whitney U post hoc tests were used to compare median ages. Further analysis was undertaken to describe the outcomes of the IGRA screening project 12 months later, using the clinical data extracted from NCIMS.

### Ethics

This study was approved by the Aboriginal Health and Medical Research Council (1093/15) with a waiver of informed consent, the North Coast NSW Ethics Committee (NCNSW HREC No LNR 121) and received a Site Specific Assessment Approval (LNRSSA/15/NCC).

## Results

Between 3 June 2013 and 27 January 2015, 2019 Aboriginal and Torres Strait Islander peoples presented to the ACCHS for a GP consultation.

A total of 135 individuals (61 males [45%] and 74 females [55%]) were screened for TB using IGRA, or 6.7% of all Indigenous-patient GP presentations in this period. We do not have data on how many patients declined IGRA screening. Overall, 24 of the 135 participants tested (17.8%) were IGRA positive; one (4.2%) was diagnosed with active TB.

Between October 2000 and February 2015, 37 epidemiologically linked TB MIRU pattern 23’3425153322 cluster cases in Aboriginal people were diagnosed in NSW; these individuals were not necessarily patients of the ACCHS.

### Gender and median age comparison

The gender and median ages of the ACCHS clinic patients, IGRA participants and patients of the TB cluster are presented in **Table 1**.

**Table 1:** Sex and median age comparison with ACCHS attendees as the reference group

-	ACCHS patients *n* = 2019	Patients screened for TB by IGRA *n* = 135	*P*-value	IGRA positive *n* = 24	*P*-value	TB cluster cases	*P*-value
Sex*	**-**	**-**	**0.740**	**-**	**0.626**	**-**	** < 0.01**
**Male**	**942 (47%)**	**61 (45%)**	**-**	**10 (42%)**	**-**	**28 (76%)**	**-**
**Female**	**1077 (53%)**	**74 (55%)**	**-**	**14 (58%)**	**-**	**9 (24%)**	**-**
Median age** (years)							
**Male**	**20**	**44**	** < 0.01**	**48**	** < 0.01**	**41**	** < 0.01**
**Female**	**24**	**43**	** < 0.01**	**49**	** < 0.01**	**41**	** < 0.01**
**Range age (years)**	**0–88**	**3–75**	**-**	**19–66**	**-**	**0–65**	**-**

* Pearson’s χ^2^ test for gender comparison ** Mann–Whitney U post hoc test for median age comparison

No statistically significant difference in sex distribution was found between ACCHS patients and patients screened by IGRA (χ^2^ = 0.11, *P* = 0.74) and IGRA participants who screened positive for LTBI (*P* = 0.63). Significantly more of the cases within the TB cluster were among men (76%) compared to all ACCHS attendees (47%, *P* ≤ 0.01), all IGRA participants (45%, *P* ≤ 0.01) and IGRA participants who screened positive for LTBI (42%, *P* ≤ 0.01).

The median ages of patients tested by IGRA (male 44 years/female 43 years; range 3 to 75 years), those who were IGRA positive (male 48 years/female 49 years; range 19 to 66 years), and those within the TB cluster (male 41 years/female 41 years; range 0 to 65 years) were significantly higher compared to the general ACCHS patient population (male 20 years/female 24 years; range 0 to 88 years; *P* ≤ 0.01 for all groups and both sexes.)

### Outcome analysis

Out of 135 IGRA tests, 102 (75.6%) were negative, nine (6.7%) were indeterminate and 24 (17.8%) were positive for TB. In this report, indeterminate test results were not included in the screening outcome analysis.

#### Positive IGRA tests

Of the 24 people who tested IGRA positive, 13 (54.2%) were newly diagnosed with LTBI and one (4.2%) was diagnosed with active TB. The other 10 (41.7%) participants with LTBI had positive TSTs before the IGRA screening documented in the NCIMS database, but the TST results were not documented in the ACCHS medical records and had not been disclosed by the participants at the time of IGRA screening.

Eight of the 24 participants who were IGRA positive (33.3%) reported having a cough; one participant reported cough, weight loss and night sweats, and one participant reported general malaise. All 24 participants who were IGRA positive were referred for chest X-ray and sputum testing for AFB.

Sixteen participants (64%) had a chest X-ray. Eleven (68.8%) were reported as normal and five (31.3%) were abnormal (reported findings were atelectasis, chronic obstructive pulmonary disease, pulmonary nodules and pleural effusion, consolidation and bilateral consolidation).

Five of the 24 participants (20.8%) had sputum tested for AFB. The sputum of one participant was smear and PCR positive for AFB and culture positive for *M. tuberculosis*. This participant had reported night sweats and weight loss, and active TB was considered at the time of presentation. The participant completed treatment for active TB.

In summary, of the 24 IGRA positive participants referred for sputum testing and chest X-ray 21 (87.5%) had a chest X-ray and/or sputum tested. One participant had chest X-ray and sputum testing resulting in a diagnosis of active TB, 16 had chest X-ray only; 4 had sputum testing only; and 3 did not have chest X-ray or sputum testing. The active TB case was the participant with TB-like symptoms and consolidations on chest X-ray.

#### 12 months follow-up

Twelve of the 14 participants (85.7%) with a new positive TB screening result (13 LTBI and 1 active TB) had a chest X-ray at the time of the positive test, and 6 of these 12 (50%) had a repeat chest X-ray at 12 months. One person died of other causes before the scheduled chest X-ray at 12 months. Four participants (40%) of the 10 who tested IGRA positive with a previous positive TST had a chest X-ray at the time of their positive IGRA test but did not have a repeat chest X-ray at 12 months.

Referral for preventive treatment and assessment of previous TB contact and counselling was offered to all patients with newly diagnosed LTBI; however, none who had LTBI detected by IGRA screening commenced preventive treatment for LTBI. The patient diagnosed with active TB completed treatment for TB.

## Discussion

This screening project involving 135 participants reached both males and females. The median age for patients tested with IGRA was 44 years for males and 43 years for females. Twenty four of the 135 (18%) screened by IGRA had positive tests. Twenty one (87.5%) of those who tested positive had chest X-ray and/or sputum testing. One case of active TB was diagnosed. None of those with positive IGRA results that were interpreted as indicative of latent TB infection initiated treatment.

Of the 2019 ACCHS presentations, 135 (7%) participated in the IGRA screening. It is unknown how many patients were offered IGRA; however, the participating GPs reported that most patients accepted it. Symptoms reported by participants can create selection bias, but we did not assess how these reports influenced GPs’ decisions to offer screening. Other biases that potentially increased screening included GP awareness of TB in the household or other TB contact (unknown to the patient), knowledge of lifestyle factors (such as smoking and drug use), the patient’s living circumstances and GP’s TB knowledge. Both providers and participants may have been influenced by their awareness of TB in local communities or the diagnosis of a case of active TB at the ACCHS. We tried to mitigate bias by providing basic TB training and TB screening instructions to all clinical staff at the ACCHS before commencement of this project. Research into what encourages provider and patient participation in TB screening in this setting is required.

The screening project reached both males and females, including men in the age group most affected by TB in the MIRU pattern 23’3425153322 cluster. (*13*) Previous informal reviews of TB screening activities by the PHU TB team displayed a disproportional low participation rate among men. We believe that the established doctor–patient relationship between the GPs offering IGRA screening to their male patients contributed to our results. Furthermore, unpublished research involving interviews with Aboriginal men affected by TB suggest TB screening in the annual Aboriginal and Torres Strait Islander Health Assessment at the ACCHS would further enhance participation. A formal comparison of outcomes from contact screening in this setting with the IGRA project would assist in adapting targets and interventions to local epidemiology as recommended by WHO. (*16*)

We found that 17.8% of the people screened were infected with *M. tuberculosis*. This result should be interpreted with caution as only 7% of the target population was screened; therefore, population prevalence cannot be inferred from this study. The median estimated population prevalence for LTBI in Australia is between 0% and 10%, which is lower than our findings. (*17*) Follow-up studies are needed to make conclusions about the TB prevalence in this community.

Access to specialized TST and LTBI follow-up services are limited for the widely dispersed rural Aboriginal communites in northern NSW. TST has proven insufficient for preventing ongoing TB transmission in these communites. We believe IGRA offered in ACCHSs with the support of a specialized TB service would further understanding of the prevalence of TB and allow screening in communities that may have high infection rates.

Medicare funding for IGRA has increased since implementation of this pilot project, but it only covers screening if a patient is a contact of an active case, even in groups with higher TB notification rates. Indigenous peoples are a “vulnerable and hard-to-reach group” as defined by WHO, and interventions must be designed to increase access to TB services. (*16*) Medicare-funded IGRA is required for ACCHSs to provide autonomous services to identify, treat and manage LTBI. The number of indeterminate IGRA test results reinforced that IGRA screening must include staff training on specimen collection, particularly on proper processing time frames and storage of specimens.

The participation rate for sputum collection for AFB, smear and culture (20.8%) might have been influenced by low rates of cough and by the logistics of sputum collection and delivery to the private pathology laboratory. On-the-spot sputum collection in the ACCHS setting and transport to the laboratory is being promoted to increase the number of people who have at least one sputum specimen tested for AFB.

Early diagnosis of active TB is a valuable tool for TB prevention. (*18*) Improving the rate of sputum collection and follow-up chest X-ray at 12 months can enhance the diagnosis of early TB. CXRs to diagnose active TB were obtained from 85.7% of the participants with newly diagnosed LTBI, and 50% repeated a chest X-ray 12 months later. Identifying and addressing Aboriginal and Torres Strait Islander peoples’ concerns regarding radiological testing may improve follow-up rates.

LTBI treatment reduces the risk of progression to active disease for high-risk individuals. (*16*) A population-based study with Indigenous populations in the United States of America, Greenland and Canada found LTBI screening and treatment were associated with significant decreases in TB notification rates. (*19*) In our study, none of the participants with newly diagnosed LTBI were treated for latent TB despite a range of patient-centred services offered by the ACCHS and the PHU TB team. Aboriginal health workers facilitated communication between the patient, GP and the PHU TB Team and provided care coordination and emotional support to the patient. Convenient appointment times during business hours, and transport to specialist appointments were also offered. It is likely that health service and patient influences were barriers to treatment for LTBI, including treatment length, presence of contraindicating medical conditions, the potential for adverse medication reactions and access to specialist TB services, which have been shown in previous studies. (*20*, *21*) Health services must elicit and be receptive to Aboriginal and Torres Strait Islanders peoples’ views on LTBI treatment and find a way forward together to prevent TB. Regularly providing TB specialist services at the ACCHS could improve follow-up, strengthen the ACCHS-TB Team partnership, increase TB knowledge in GPs, and contribute to two-way learning with Aboriginal and Torres Strait Islander peoples.

Since the start of the pilot project, many Aboriginal and Torres Strait Islander people have requested further information on TB or referred someone they knew with a history of TB to the ACCHS. The PHU TB team, respiratory medical specialists and GPs with TB knowledge and experience continue to provide outreach and education support for TB to ACCHS staff, including the doctors working in this community.

### Limitations

We could not estimate LTBI prevalence because only 7% of the target population was screened. Financial support for the IGRA tests was limited and we were unable to continue to offer screening. The study design with a discretionary, GP-led approach to offering IGRA screening does not allow any conclusions regarding the reasons for or against participation by the patient. Furthermore, we are unable to comment on the reasons or restraints to offering IGRA by the GP. A structured approach for IGRA screening (for example in the annual health assessment) as well as documented reasons as to why a patient declined the offered screening will improve evaluations of this model of care. Limited data were available to compare participant characteristics. This study was not able to compare the IGRA screening outcomes with those for routine TB screening methods.

## Conclusion

TB incidence for the Australian Aboriginal and Torres Strait Islander population is significantly higher than the Australian-born non-Indigenous population, (*2*) and accessible and socioculturally appropriate health services are required to support the unique structures and care needs of Aboriginal communities. Two of the main limiting factors of this pilot project were the costs of IGRA and a discretionary approach to screening. Further research, with a structured approach, needs to further evaluate the effectiveness of this model of care. We recommend that IGRA becomes accessible under Medicare for Aboriginal and Torres Strait Islander people and for TB screening to be incorporated into the annual health assessment as a routine screening.

Ongoing community engagement and collaboration is necessary to develop TB elimination strategies for vulnerable and hard-to-reach groups. Increasing access to screening and specialist care through the ACCHS will support this. This project identified policy and practice issues that need to be addressed to implement a sustainable TB screening programme with IGRA in an ACCHS.
